# Hemodynamics-driven mathematical model of third heart sound generation

**DOI:** 10.3389/fphys.2022.847164

**Published:** 2022-10-11

**Authors:** Mehrdad Shahmohammadi, Wouter Huberts, Hongxing Luo, Philip Westphal, Richard N. Cornelussen, Frits W. Prinzen, Tammo Delhaas

**Affiliations:** ^1^ Department of Biomedical Engineering, Cardiovascular Research Institute Maastricht (CARIM), Maastricht University, Maastricht, Netherlands; ^2^ Department of Physiology, Cardiovascular Research Institute Maastricht (CARIM), Maastricht University, Maastricht, Netherlands; ^3^ Bakken Research Centre, Medtronic, BV, Maastricht, Netherlands

**Keywords:** third heart sound, mathematical modelling (medical), cardiac vibration, heart sound (HS), pathological heart sound

## Abstract

The proto-diastolic third heart sound (S3) is observed in various hemodynamic conditions in both normal and diseased hearts. We propose a novel, one-degree of freedom mathematical model of mechanical vibrations of heart and blood that generates the third heart sound, implemented in a real-time model of the cardiovascular system (CircAdapt). To examine model functionality, S3 simulations were performed for conditions mimicking the normal heart as well as heart failure with preserved ejection fraction (HFpEF), atrioventricular valve regurgitation (AVR), atrioventricular valve stenosis (AVS) and septal shunts (SS). Simulated S3 showed both qualitative and quantitative agreements with measured S3 in terms of morphology, frequency, and timing. It was shown that ventricular mass, ventricular viscoelastic properties as well as inflow momentum play a key role in the generation of S3. The model indicated that irrespective of cardiac conditions, S3 vibrations are always generated, in both the left and right sides of the heart, albeit at different levels of audibility. S3 intensities increased in HFpEF, AVR and SS, but the changes of acoustic S3 features in AVS were not significant, as compared with the reference simulation. S3 loudness in all simulated conditions was proportional to the level of cardiac output and severity of cardiac conditions. In conclusion, our hemodynamics-driven mathematical model provides a fast and realistic simulation of S3 under various conditions which may be helpful to find new indicators for diagnosis and prognosis of cardiac diseases.

## Introduction

The third heart sound (S3) is a low-frequency early diastolic sound that occurs during rapid filling of the ventricles under several physiological and pathological states (Van de Werf et al., 1984a; Mehta and Khan, 2004). In healthy young adults, S3 is distinguishable during exercise and diminishes with aging (Van de Werf et al., 1984a; Mehta and Khan, 2004). In patients, the existence of an audible S3 may be symptomatic for conditions such as heart failure with preserved ejection fraction (HFpEF), atrioventricular valve regurgitation (AVR) and septal shunts (SS) (Van de Werf et al., 1984a; Mehta and Khan, 2004).

Various studies have provided scattered information about the relationship between hemodynamics, anatomical factors and acoustic features of S3 in physiological and pathological states. Previous works each have focused only on a few subsets of the factors and conditions associated to S3. For instance, in physiological states, Van de Werf et al. (1984b) focused on aging-induced hypertrophy which decreases S3 amplitude, while Zhong et al. (2011) showed that exercise increases amplitude and frequency of S3. In pathological states, Folland et al. (1992) concluded that S3 is more frequent in atrioventricular regurgitations than in stenotic lesions. They also showed that severity of regurgitation affects S3 and makes it more distinguishable (Folland et al., 1992). Patel et al. (1993) related the presence of S3 in mitral regurgitation to increases in end-diastolic volume (EDV) and regurgitant fraction. In addition, patients with heart failure may have S3 because of low ventricular compliance, elevated filling pressures, or increased early diastolic filling rates (Gazewood and Turner, 2017; Gheorghe et al., 2019; van Loon et al., 2020).

Most researchers have related S3 to intraventricular blood flow velocity in early diastole (E wave). Various theories have been postulated concerning main structures that vibrate due to this rapid flow: Valvular theory (Dock et al., 1955) (a vibration generated by transient partial closure of the mitral valve), impact theory (Redddy et al., 1981) (the heart hits the chest wall and generates S3 vibrations), and ventricular theory (Van de Werf et al., 1984a) (a rapid inrush of blood sets the ventricular wall in conjunction with the blood mass in vibration since the blood and ventricular walls are in contact).

Computational models can help to further understand the mechanisms behind the generation of S3 and provide some insights into these disputes. Several studies (Held et al., 1984; Drzewiecki et al., 1991; Longhini et al., 1996) constructed models based on the valvular and impact theories, but were then refuted experimentally (Mehta and Khan, 2004). The widely accepted ventricular theory has neither been disproved nor fully corroborated (Mehta and Khan, 2004).

**FIGURE 1:**
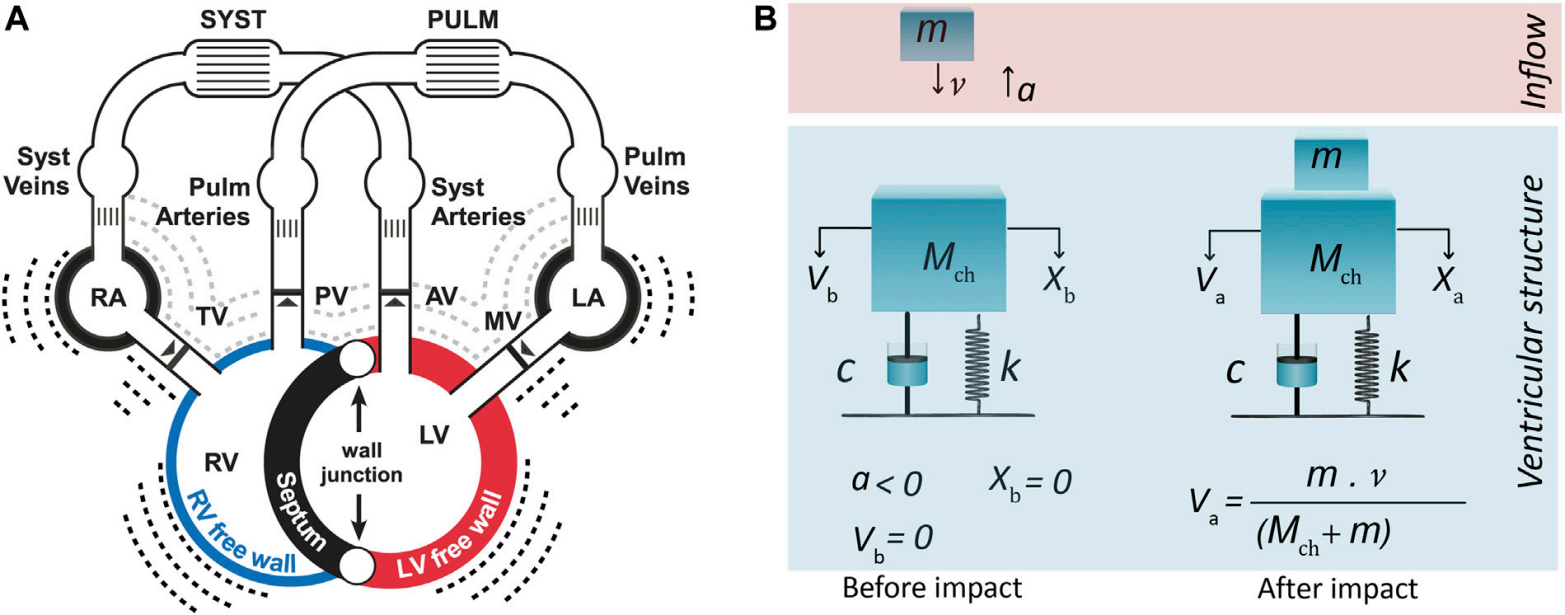
**(A)** CircAdapt model for simulation of cardiovascular mechanics and hemodynamics of the entire circulation (Walmsley et al., 2015). **(B)** One-degree-of-freedom model for third heart sound generation embedded in CircAdapt. *The model before and after impact of blood inflow from atrium.* Inflow blood mass (*m*) hits ventricular cardiohemic mass (*M*_*ch*_) and imparts momentum (*m·* v ) to the cardiohemic structure. Panel (a): LA, left atrium; LV, left ventricle; MV, mitral valve; PV, pulmonary valve; RA, right atrium; RV, right ventricle; TV, tricuspid valve. Panel (b): a, acceleration of inflow blood before impact, c, damping factor; k, spring factor; $M_{\mathrm{ch}}:$ *Ventricular* cardiohemic mass (was obtained by summation of the CircAdapt-derived chamber wall (*m*_*myo*_) and blood volumes (*m*_*bld*_))*, m*, inflow blood mass; v , velocity of inflow blood mass before impact; $V_{b}$ , velocity of ventricular cardiohemic mass (*M*_*ch*_) before impact; $V_{a}$ , velocity of ventricular masses (*M*_*ch*_ *+ m*) after inelastic impact; *X*_*a*_, movement of ventricular masses (*M*_*ch*_ *+ m*) after impact; *X*_*b*_, movement of ventricular cardiohemic mass (*M*_*ch*_) before impact.

In the present study, we propose a ventricular theory-based vibration model of S3 generation integrated into CircAdapt (Figure 1A) which is a closed-loop lumped parameter model of the cardiovascular system. CircAdapt can simulate cardiac strain patterns, blood pressures, flows and volumes in both normal and pathophysiological conditions (Walmsley et al., 2015). In a previous study we extended CircAdapt with an S1/S2 module (Shahmohammadi et al., 2021), now we want to add an S3 module. The simulated hemodynamics of normal and pathophysiological conditions in CircAdapt were used to generate initial conditions for a one-degree-of-freedom mass-dashpot-spring model which mimics the visco-elastic mechanics of cardiohemic structures. The constructed model was applied to investigate hemodynamic/anatomic causal relations to S3 acoustic features in both physiological (aging and exercise) and pathological states (AVR, SS, HFpEF) compared to conditions with lower audible S3 (atrioventricular valve stenosis (AVS), normal).

## Methods

### S3 model

The genesis of S3 sounds in the ventricular theory (Van de Werf et al., 1984a) is built on considering the dynamics of the ventricular cardiohemic system, i.e., ventricular blood flow and myocardial masses in conjunction with myocardial viscoelasticity. The theory assumes that the impact of blood inflow at peak atrioventricular blood velocity, where deceleration of mitral and tricuspid flow starts, induces/causes the onset of the first vibration of S3.

During ventricular diastole, initially ventricular pressure drops below atrial pressure and then increases due to passive ventricular filling. The increase in left ventricular pressure causes a deceleration of the valvular inflow (Van de Werf et al., 1984a). At the beginning of deceleration, when blood inflow is peaked and atrioventricular pressure differences becomes zero, we can assume that no external force acts on the system and that only the existing momentum of the blood flow impacts the system. Hence, the system at this time point can be considered as a free vibrating system which is put out of the temporal equilibrium state only by blood impact.

We can now mimic the above-mentioned free vibration of the cardiohemic system with a single-degree-of-freedom lumped parameter model (Figure 1B). The structure of the model comprises of one single cardiohemic mass (*M*_ch_), which is a combination of ventricular cavity blood mass, *m*_*bld*_, and ventricular free wall myocardium mass, *m*_*myo*_, connected to two linear time-dependent mechanical elements (i.e., a spring with stiffness k(t) and a dashpot with damping factor c(t)). The resulting governing equation for the free vibration is given by:

$$\left({\text{M}}_{\text{ch}}\right)\;\ddot{x}\left(t\right)+c\left(t\right)\dot{x}\left(t\right)+k\left(t\right)x\left(t\right)=0,$$ (1)

in which *x* is displacement of the masses.

The model is put out of equilibrium state by the impact of inflowing blood from the atrium to the ventricle at a free vibration moment when pressure difference across the atrioventricular valves is zero (Figure 1B). Hence, *x(t)* represents the vibration of the lumped cardiohemic mass induced by the impact of blood. To solve the above differential equation, we need a model to simulate the impact of blood and proper expressions for the spring and dashpot elements. Moreover, we need a model that can simulate blood velocities and pressures. S3 generation in the left and right ventricle was simulated by using the same type of differential Eq. 1 but with different parameters.

### Model of blood impact

Incoming blood mass (*m*) with certain velocity (*v*) imparts momentum (*m·v*) to the cardiohemic structure. The impact is considered as inelastic collision of two masses. Therefore, after impact, inflowing blood mass (*m*) blends with the existing cardiohemic lumped mass and thereby increases the amount of blood existing in the ventricle.

Since we assumed that there are no external forces working on the system, the momentum before and after collision of incoming blood mass should be conserved. This leads to:

$$\left({\text{M}}_{\text{ch}}+\text{m}\right)V_{a}-\left(m\cdot \text{v}+{Μ}_{\text{ch}}\cdot V_{b}\right)=0,$$ (2)

in which $V_{\text{a}}$ is the velocity directly after the impact of the incoming blood mass (m) blended with the cardiohemic lumped mass *M*_*ch*_, $V_{\text{b}}$ is the initial velocity of the cardiohemic mass before impact (which is zero due to the equilibrium state assumption of the system), and v  is the mean velocity of inflowing blood mass before impact (calculated real-time by flow rate through the valves and their opening area). The velocity $V_{\text{a}}\;$ can now be derived from Eq. 2. This gives:

$$\;V_{\text{a}}=\frac{m\;\cdot \;v}{\left(M_{\mathrm{ch}}+m\right)}.$$ (3)

Since the impact makes an immediate change in the velocity, but not the displacement, the initial conditions of Eq. 1 can be considered as:

$$x\left(t=0\right)=0\;\mathrm{and}\;\dot{x}\left(t=0\right)=V_{\text{a}}.$$ (4)

Now we can find the analytical solution of Eq. 1, which reads:

$$x\left(t\right)=\frac{m\;\cdot v}{\left(M_{\mathrm{ch}}+m\right)\;\omega_{\text{d}}}\;e^{-\zeta \omega_{\text{n}\;}t}\sin\omega_{\text{d}}t.$$ (5)

in which $\zeta ,\;\omega_{\text{n}\;},\;\omega_{\text{d}}$ are damping ratio, natural resonance frequency, and damped resonance frequency respectively of the lumped system for a time point. They are defined by:

ζ= c2k(Mch+m) (6)

ωn = k(Mch+m) (7)

$$\omega_{\text{d}\;}=\;\omega_{\text{n}\;}.\;\sqrt{\left(1-\zeta^{2}\right)}$$ (8)

### Estimation of model parameters and variables

In addition to a blood impact model, proper expressions are needed for $M_{\mathrm{ch}},\;\;m,\;v,\;k,\;c$ at each time snapshot before the S3 sound can be simulated. Because CircAdapt (Figure 1A) proved to be a reliable software for simulation of cardiovascular mechanics and hemodynamics in various conditions (Walmsley et al., 2015; Shahmohammadi et al., 2021), we used this software to identify the most appropriate values for our model parameters. Details about the used parameters of CircAdapt are extensively described in (Walmsley et al., 2015; van Loon et al., 2020). The ventricular cardiohemic ( $M_{ch}$ ) mass is defined as the summation of *m*_*myo*_(chamber free wall volume multiplied by its density 1,055 kg/m^3^ (Gheorghe et al., 2019)) and *m*_*bld*_ (blood volume trapped in ventricle multiplied by 1,050 kg/m^3^), *m* was estimated by integrating the volume flow rate across the valves (Q) over a fixed 1 ms time interval (i.e., one snapshot) and subsequently, multiplying the resulting volumes with blood density 1,050 kg/m^3^, *v* by flow rate across the valves divided by their respective cross-sectional area, values of spring factor *k* (derived from myocardial stiffness) and of damping factor *c* were calibrated by comparison of S3-vibrations between simulation and recording (from PhysioNet dataset (Liu et al., 2016)) in order to have the same peaks for a healthy subject with physiological S3. The amount of damping factor was estimated as 10 percent of spring factor. The S3 generation model was activated at a free vibration moment when pressure difference across the atrioventricular valves was zero. Finally, the final S3 is addition of the S3 generated in each ventricle separately.

### Integration into CircAdapt

CircAdapt was simulated for various cardiac conditions. After reaching steady-state solution for each condition, the resulting cardiovascular mechanical and hemodynamic variables were fed into our acoustic module at each cardiac cycle real-time. The module consisted of two models: S1/S2 and S3. Since these models are based on different theories and deal with different phases within the cardiac cycle, they were run separately but the results were added in order to make the final heart sound. The starting time for our models were different. The starting time for S3 model is corresponding to peak E-wave and we considered no vibration caused by S3 before this time. But there are vibrations caused by S1 and S2. Hence, we added S3 to S1-S2 vibrations without interference in the occurrence times and, consequently, without interference in the signals. The input parameters ( $M_{\mathrm{ch}}\;(=m_{\text{bld}}+m_{\text{myo}}),m,\;v,\;a)\;$ of the S3 model were calculated from the variables in CircAdapt at peak E-wave to calculate the output which is vibration (*x*).

### Simulation protocol

Model functionality was tested under physiological and pathological states which are known to have detectable (i.e. AVR, SS, HFpEF) and non-detectable (i.e. AVS, normal) S3 (Van de Werf et al., 1984a; Mehta and Khan, 2004). We simulated cardiac conditions using CircAdapt for a healthy adult at rest (reference) as well as during exercise, and for various disorders: HFpEF, AVR, AVS, SS. Simulated S3 was characterized by acoustic features which comprises frequency, timing and morphology of the signal.

Homeostatic pressure-flow regulation was applied to all simulations and was achieved by modulation of both the systemic peripheral vascular resistance and circulating blood volume to maintain mean arterial pressure at 91 mmHg and cardiac output (CO) at each level of exercise intensity during the adaptation process (van Loon et al., 2020).

### Simulation conditions

**Exercise:** Exercise was simulated by increasing heart rate (HR) from 70 to 130 (beat/min) and CO from rest (5 L/min) to exercise (14 L/min) in steps of 3 L/min. The SV (stroke volume)-CO relationship is based on measurements from healthy non-trained adults (Lumens et al., 2019).

**Aging:** The effects of aging on cardiac function were simulated in CircAdapt by increasing both decay time of myocardial contractility and myocardial stiffness from 2% to 12% of their reference values (with steps of 2%).

**SS:** Atrial septal defect (ASD) and ventricular septal defect (VSD) were simulated by creating an orifice in the atrial and ventricular septum, respectively, from 6 to 12 mm with steps of 2 mm.

**AVR:** Mitral regurgitation (MR) and tricuspid regurgitation (TR) were simulated by leaving 8–14% (at steps of 2%) of the area of the corresponding valves open during ventricular contraction.

**AVS:** For mitral stenosis (MS) and tricuspid stenosis (TS) simulation, the opening area of the corresponding valve was narrowed from 50% to 80% at steps of 10%.

We estimated the severity of all pathological conditions (SS, AVR and AVS) based on the level of leakage/obstruction.

**HFpEF:** The following two LV diastolic abnormalities associated with HFpEF were imposed on the septal and LV free walls to evaluate their effects on cardiovascular system dynamics (van Loon et al., 2020) (Table 1):

• Impaired LV relaxation function: the time constant of LV isovolumic relaxation (Tau) was increased.
• Increased LV passive stiffness: the end-diastolic elastance (Eed) was increased.

**TABLE 1:** Simulation protocol for three severity levels of HFpEF. HFpEF, heart failure with preserved ejection fraction; Eed, end-diastolic elastance; Tau, time constant of LV isovolumic relaxation.

HFpEF	Grade Ι impaired relaxation	Grade ΙΙ Pseudonormal	Grade ΙΙΙ restrictive
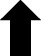 Tau (% )	80	80	80
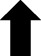 Eed (% )	0	75	130

In all above simulations except in exercise, CO and HR were maintained at 5 L/min and 70 beats per minute, respectively.

### Phonocardiogram data

To compare simulated S3 for each cardiac condition with recordings on the chest, we analyzed frequency, timing and morphology of the most noise-free recording for the same condition found in PhysioNet heart sound dataset (Liu et al., 2016), an open-access heart sound database of various cardiac conditions with and without S3, collected for an international competition. Recordings with S3 were collected, resampled to 1,000 Hz, and band-pass filtered between 25 and 400 Hz. Finally, any spikes in the recording results were removed.

### Integrated loudness

Integrated loudness, a measure proposed by EBU R 128 standard (see Appendix A ) to measure the amount of loudness perceived by human ears throughout the whole duration of a soundtrack, was used to show changes in loudness of S3 in different conditions.

## Results

**FIGURE 2:**
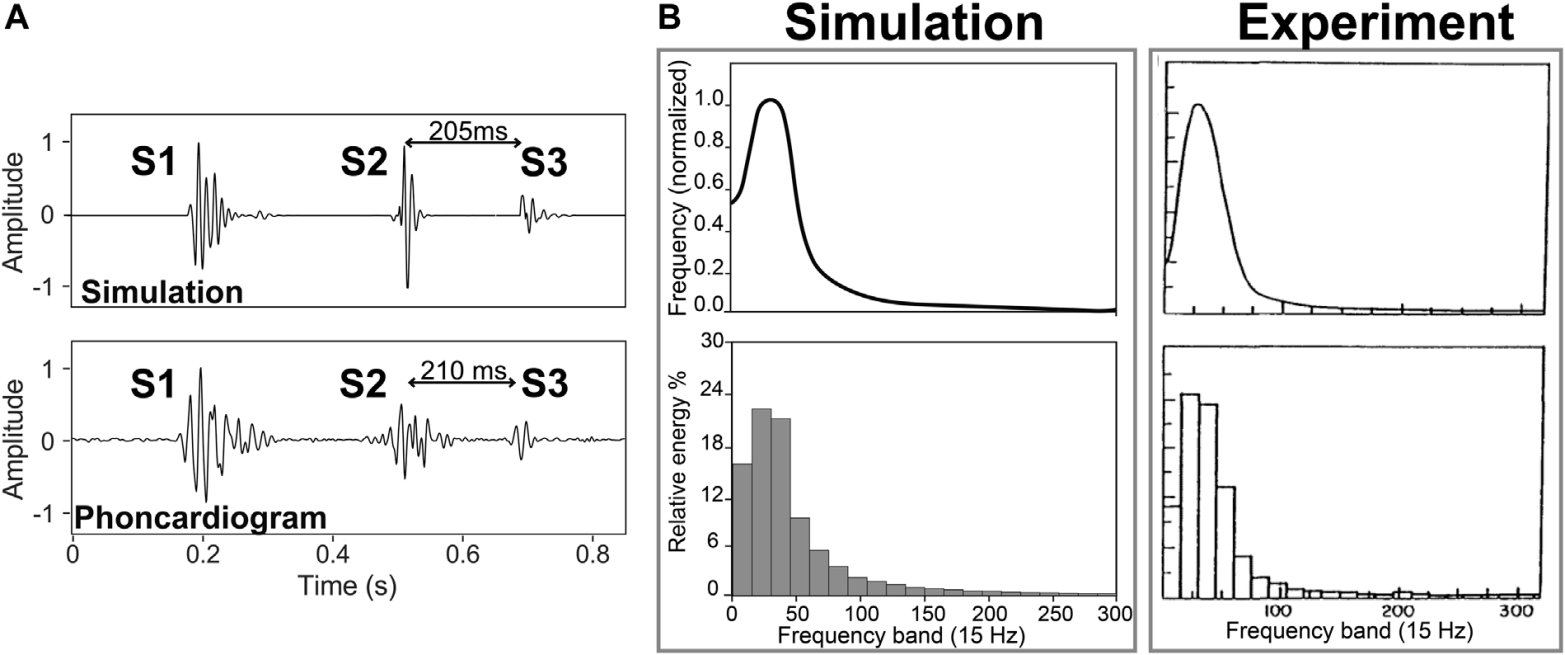
**(A)** S3 location in the experiment (Liu et al., 2016) and simulation of a healthy subject with a cardiac output of 5 L/min. **(B)** Frequency spectrum and normalized energy of S3 from experiment (Longhini et al., 1996) and simulation (5 L/min).

**Morphology.** Figure 2A compares time of occurrence, duration, and frequency of S3 obtained from phonocardiogram and simulation. The phonocardiogram is from a healthy subject with S3 in PhysioNet dataset (Liu et al., 2016). The simulation is composed of simulated first and second heart sounds from a previous study (Shahmohammadi et al., 2021) superposed to the simulated S3 in normal condition at rest (5 L/min)). Note the close agreement between the phonocardiogram and the simulation.

**Frequency.** Figure 2B shows frequency spectrum and relative energy of S3 from experiment (Longhini et al., 1996) and simulation for a healthy subject (with a cardiac output of 5 L/min). Signals were lowpass-filtered with a cutoff frequency of 300 Hz. Using a trapezoidal technique, the area under the corresponding spectrum for each band (15 Hz) was calculated as an index of the energy of the band and was normalized using the total energy. The results showed a monophasic curve with around 3/4 of the total energy below 60 Hz and with a dominant frequency around 27 and 28 Hz in simulation and experiment, respectively.

**FIGURE 3:**
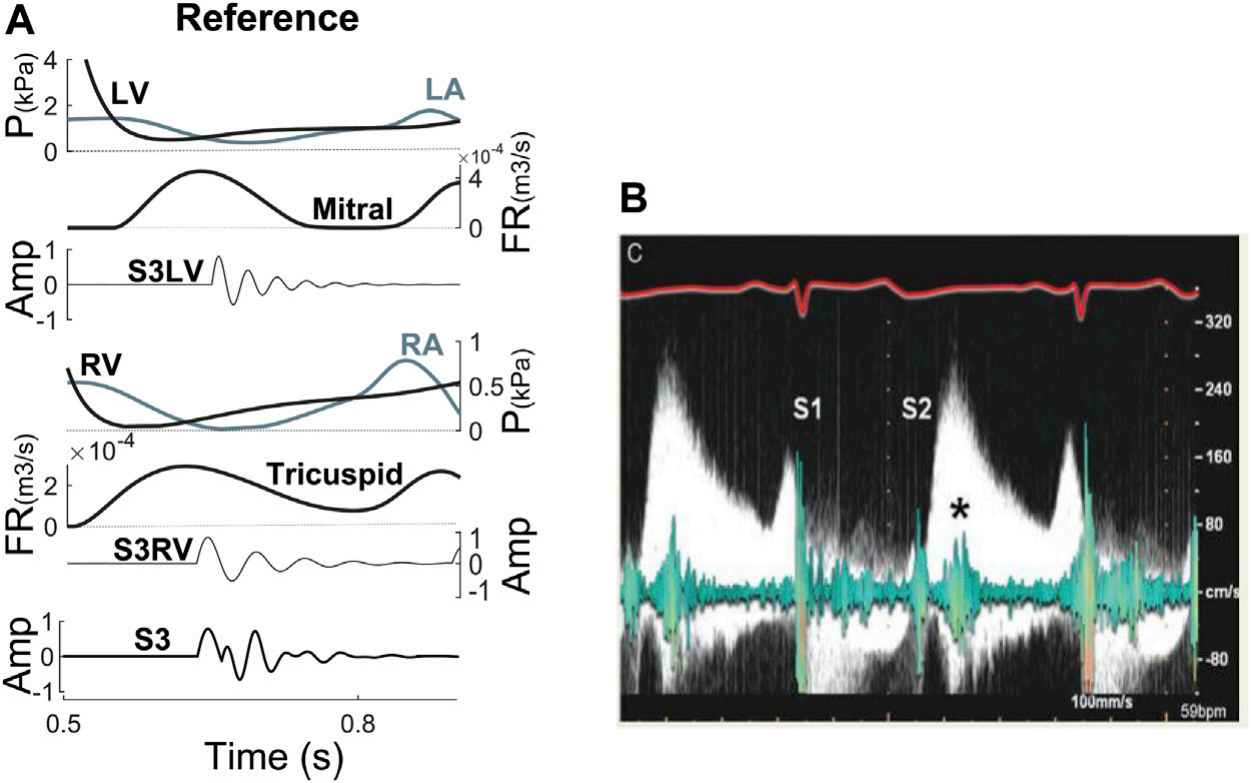
**(A)** S3 relationship with hemodynamic factors at reference. **(B)** Relation between E wave from Doppler echocardiography and S3 from phonocardiogram (Miranda et al., 2015). Amp, amplitude; FR, flow rate; LA, left atrium; LV, left ventricle; P, pressure; RA, right atrium; RV, right ventricle; S3LV, left ventricular S3; S3RV, right ventricular S3.

**Timing.** Figure 3A illustrates the relation between the simulated S3 and hemodynamics (pressures, volumes, E and A wave) of one cardiac cycle. For comparison, Figure 3B shows the relationship between S3 and E and A wave from echocardiography (a 70-year-old woman with MR) (Miranda et al., 2015). Note the coincidence between S3 occurrence and peak E wave velocity.

### Parameter Analysis

**FIGURE 4:**
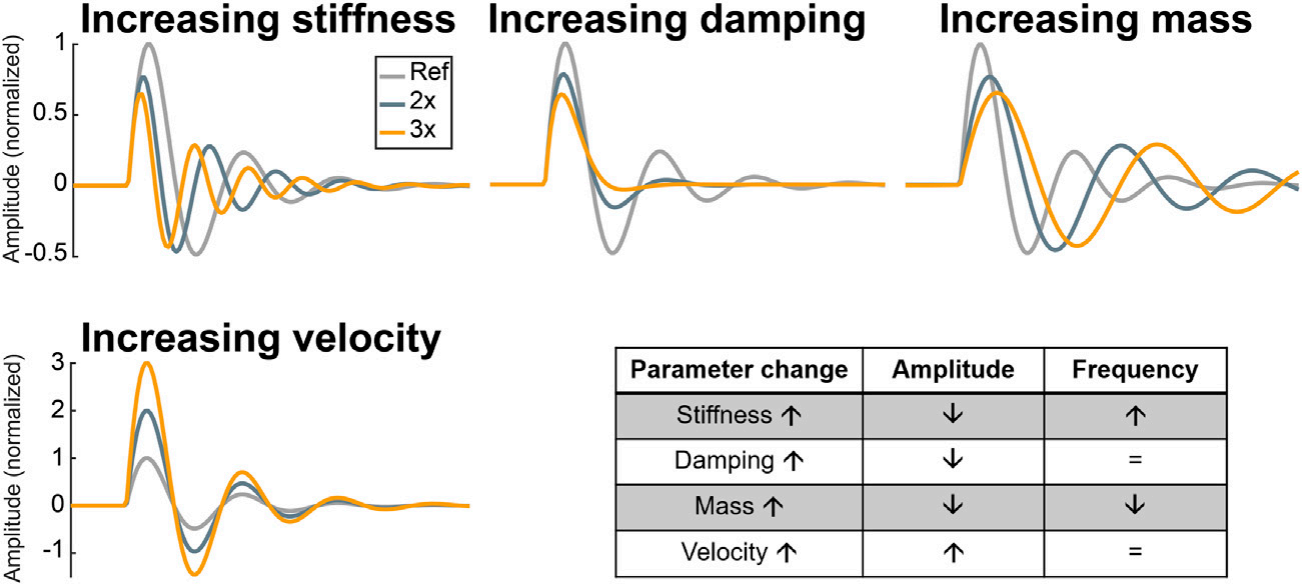
Parameter analysis of the S3 model. Ref: reference, 2x: two-fold of reference, 3x: three-fold of reference.

Figure 4 shows parameter analysis of the S3 model. It is used to better understand the behaviour of the model under physiological and pathological conditions. Separate increases in stiffness, damping or mass resulted in amplitude reduction of the generated S3, whereas increases in blood flow velocity had an opposite effect. Moreover, only two parameters were found to change the frequency of S3: ventricular stiffness and ventricular cardiohemic mass $\left(M_{\mathrm{ch}}\right)$ . The former caused an increase and the latter a decrease in frequency.

### Cardiac conditions

#### Physiological S3

**FIGURE 5:**
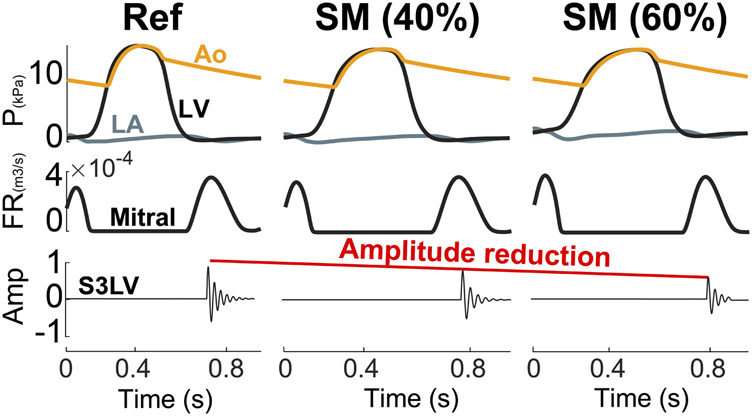
Aging causes a reduction in S3 intensity. Amp, amplitude; Ao, aorta; FR, flow rate; LA, left atrium; LV, left ventricle; P, pressure; SM, stiffer myocardium.

**Aging.** As shown in Figure 5, increasing LV stiffness in the model not only decreased S3 amplitude, but also significantly delayed its appearance in diastole. The latter was due to the delayed E wave caused by higher ventricular cavity pressure and stiffness as well as longer myocardial relaxation time.

### Pathological conditions

**FIGURE 6:**
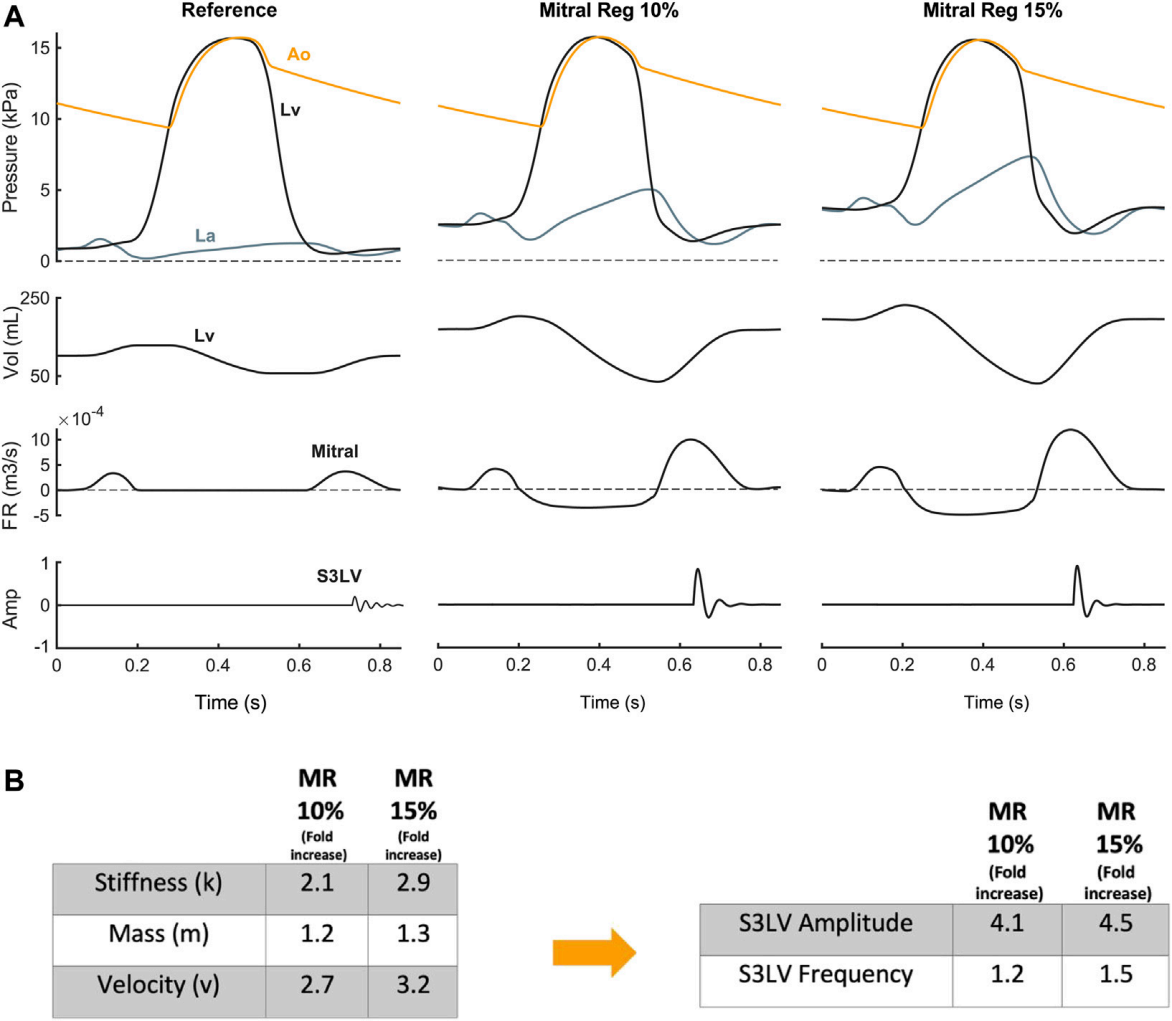
**(A)** S3 generation in mitral regurgitation. **(B)** Mitral regurgitation-induced parameter changes compared with reference and their effect on left ventricular S3 (S3LV) amplitude and frequency.

**FIGURE 7:**
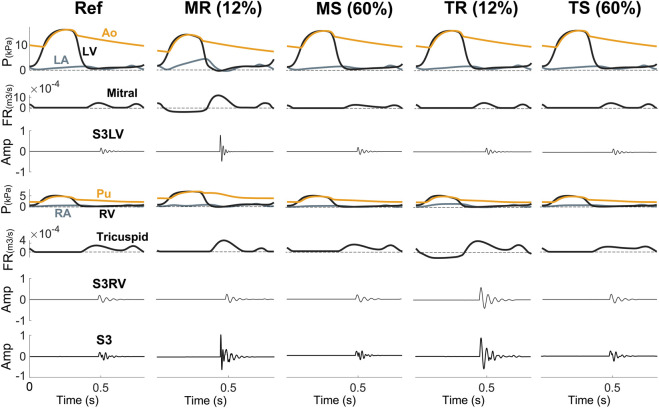
S3 is more common in disorders with leakage (MR, TR) than in stenotic lesions (MS, TS). Amp, amplitude; Ao, aorta; FR, flow rate; LA, left atrium; LV, left ventricle; MR, mitral regurgitation; MS, mitral stenosis; P, pressure; Pu, pulmonary artery; RA, right atrium; Ref, reference; RV, right ventricle; S3LV, left ventricular S3; S3RV, right ventricular S3; TR, tricuspid regurgitation; TS, tricuspid stenosis.

**Valvular diseases.** MR and TR caused stiffer myocardium, larger ventricular volume, and augmented early diastolic inflow, resulting in amplitude augmentation of left ventricular S3 (S3LV) compared to reference (Figures 6, 7). Moreover, stiffer myocardium increased the frequency of S3LV. Amplitude and frequency of both S3LV and S3RV were intensified with increasing severity of regurgitation. In contrast, MS and TS (Figure 7 MS & TS) did not change the model parameters significantly and, hence, did not lead to significant S3 changes compared to reference.

**FIGURE 8:**
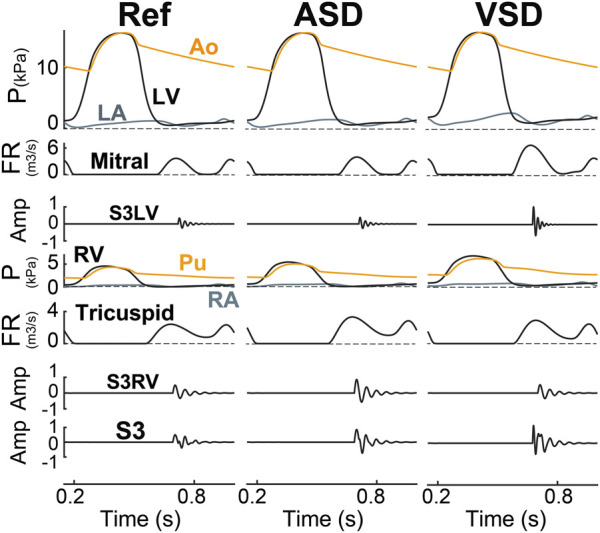
S3 as generated in ASD (6 mm) and VSD (6 mm). Amp, amplitude; Ao, aorta; ASD, atrial septal defect; FR, flow rate; LA, left atrium; LV, left ventricle; P, pressure; Pu, pulmonary artery; RA, right atrium; Ref, reference; RV, right ventricle; S3LV, left ventricular S3; S3RV, right ventricular S3; VSD, ventricular septal defect.

**Septal Shunts.** In ASD (Figure 8 ASD), increased flow across the tricuspid valve resulted in a higher momentum of blood flowing into the RV. This increased momentum in the model had an augmentation effect on the amplitude of S3RV. The increased stiffness of the myocardium increased frequency but the enlarged RV blood mass had a negative effect on that. On the other hand, due to higher ejection of RV and inflow in LA, LA pressure is on average a bit increased resulting in a slightly high E-wave across the mitral which resulted in a slight amplification in S3LV, though ignorable compared to S3RV. Similarly, in VSD (Figure 8 **VSD**), the shunt increased both the flow rate over the mitral valve and the momentum of blood mass entering LV. As the LV had a higher preload, the LV wall became stiffer. All these hemodynamic changes gave rise to higher amplitude and frequency of S3LV.

**FIGURE 9:**
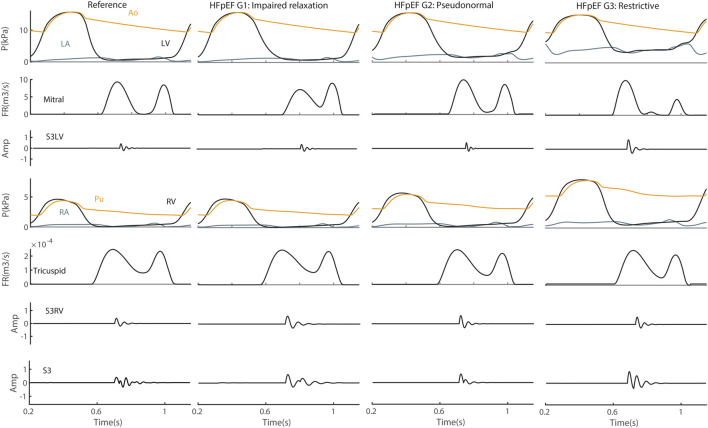
S3 in heart failure with preserved ejection fraction (HFpEF). Amp, amplitude; Ao, aorta; FR, flow rate; G1, grade 1; G2, grade 2; G3, grade 3; LA, left atrium; LV, left ventricle; P, pressure; Pu, pulmonary artery; RA, right atrium; RV, right ventricle; S3LV, left ventricular S3; S3RV, right ventricular S3.

**Heart Failure.** S3 was simulated for three grades of HFpEF, e.g., impaired relaxation, pseudonormalization and restrictive filling (Figure 9). The results indicated that both amplitude and frequency of S3LV were increased in HFpEF grade 3 compared to grade 1 and to control. There was no markedly change in the RV component of S3 in terms of amplitude and frequency.

### Loudness Index

**FIGURE 10:**
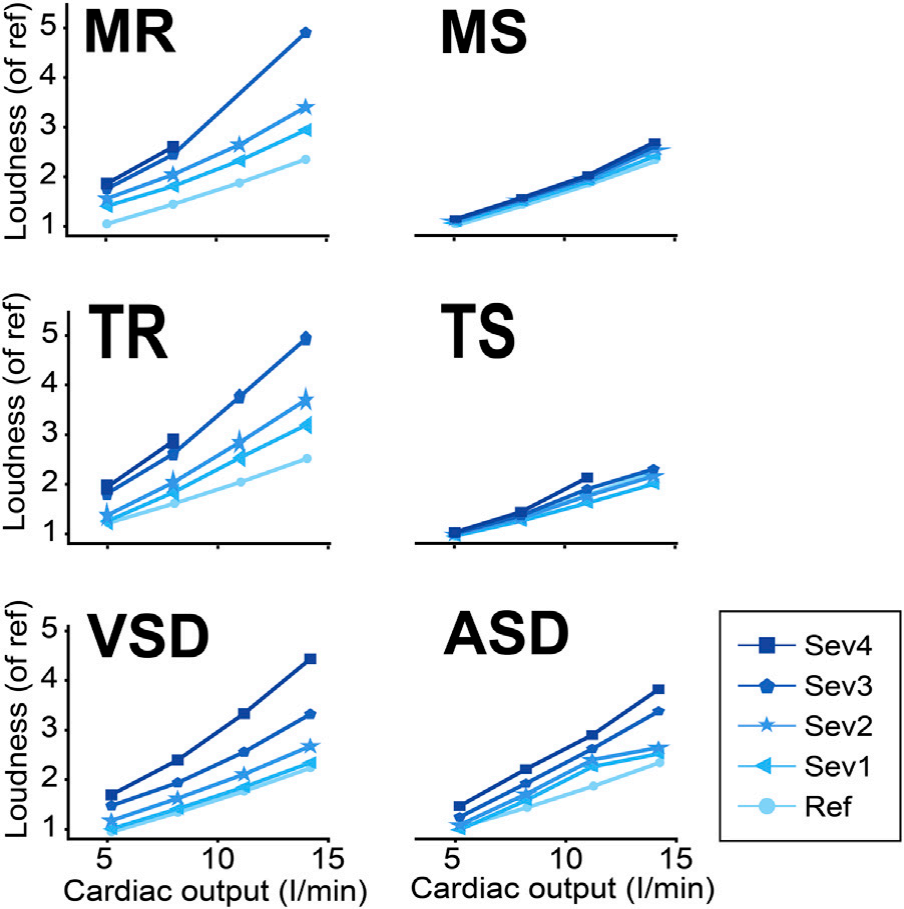
S3 loudness in various conditions and exercise levels. ASD, atrial septal defect; MR, mitral regurgitation; MS, mitral stenosis; Ref, reference; Sev 1–4, severity levels; TR, tricuspid regurgitation; TS, tricuspid stenosis; VSD, ventricular septal defect.

In all simulated cardiac conditions, S3 loudness increased with increasing exercise levels (Figure 10). Whereas S3 loudness also increased with disease severity in regurgitant and shunt conditions, stenosis severity did not markedly influence S3 loudness.

## Discussion

To the best of our knowledge, this is the first simulation study that uses an S3 generator module embedded in a complete real-time model of the cardiovascular system capable of simulations of various cardiac conditions. The results show that our model produces S3 with acoustic features that are in agreement with experimental and clinical observations.

We found that ventricular mass, inflow momentum, and viscoelastic properties play key roles in S3 generation, and that these parameters change under physiological and pathological conditions resulting in S3s with acoustic features unique to that condition in terms of frequency, timing and amplitude.

Among various vital signals used for diagnosis and prognosis of cardiac diseases, heart sounds and specifically S3 cannot be employed to their full extent because physicians subjectively assess their auscultatory findings. On the other hand, recorded heart sounds are also difficult to quantitatively relate to different cardiac conditions given the complex nature of their generation. We therefore employed cardiovascular modeling and developed a novel hemodynamics-driven mathematical model for heart sound generation to unravel the relationships between heart sounds and other vital signals. In addition, the physics-based S3 simulations in combination with flow, volume and pressure signals may help junior doctors to both improve their understanding of cardiac auscultations as well as their cardiac auscultation skills. Clinician educators are encouraged to use this strategy in addition to cardiology rotation training (Lumens et al., 2019). CircAdapt has already a user-friendly interface which is used for teaching hemodynamic signals interaction in cardiovascular system (see www.circadapt.org). Our model can be imbedded in this software as an acoustic module to help medical students with auscultation.

In regurgitant lesions (MR and TR), changes in S3 were larger than in stenotic lesions (MS and TS). Our simulation study showed that this was caused by two main factors: augmented and faster mitral inflows due to higher atrial pressure (causing an increased S3 amplitude) and increased myocardial stiffness due to a higher preload (causing an increased S3 frequency). These findings were consistent with previous studies that conditions with higher mitral inflow rates (E wave velocity) are more likely to have an audible S3 (Van de Werf et al., 1984a; Van de Werf et al., 1984b; Cowie et al., 2000).

Our model showed that S3 exists in both physiological and pathological cardiac conditions. In addition, conditions known for a right ventricular S3 (TR and ASD) also generate S3LV but at a lower amplitude and frequency (Cowie et al., 2000). We infer that in reality, the S3 with poor audibility does exist but might not have sufficiently high amplitude and/or frequency to be discriminated from background noise using human ears. Held et al. (1984) performed a study to investigate how alterations of S3 characteristics affect audibility of S3. They explained that the audibility of S3 was improved at higher amplitude and frequency. To quantify S3 audibility, we suggested to use an integrated loudness indicator which appeared to work well in our simulation study. The integrated loudness varied by disease severity and cardiac output levels. For example, the model showed that for all severity levels of MS, the loudness of S3 was lower than in MR or VSD.

Our simulations for various conditions indicate that higher ventricular peak inflows and more compliant ventricles are associated with a higher amplitude of S3 and thus better audibility of S3 whereas a larger mass is linked with poorer audibility. Our findings are in line with the study by Wilken et al. (1989) who showed that subjects without an audible S3 have heavier LV masses, lower peak E-wave velocities, and reduced rates of deceleration of early diastolic filling. However, in all HFpEF grades, increased early mitral inflow velocity increases S3 amplitude whereas increased myocardial stiffness decreases S3 amplitude. Our findings are in line with reports that patients with an S3 are more likely to have HFpEF (Mann, 2008; Gazewood and Turner, 2017).

**Comparison with other heart sound models.** Our model is integrated into CircAdapt, a well-validated complete model of the cardiovascular system allowing to generate both S3LV and S3RV in realistically simulated (pathological) cardiac conditions, while previous studies were mostly focused on S3LV in one condition without integration of the sound-generation model in a complete circulation system. In contrast to other studies, we were able to discuss how S3 changed in terms of amplitude, frequency, and timing under various common conditions and how they affect S3 loudness (Arevalo et al., 1964; Held et al., 1984; Drzewiecki et al., 1991; Longhini et al., 1996).

**Limitations**. Firstly, in the present study, a few simplifications were imposed to make the computations straightforward and to achieve a fast simulation. We employed a simple one-degree-of-freedom mechanical vibration model to simulate S3 generation. We did not model the anatomy and behavior of any individual cardiac structure because our goal was mathematical modeling of the kinematic/mechanical vibration of the ventricular cardiohemic system as a whole. We considered the cardiohemic system in each ventricle as the vibratory structure, because the idea of pure myocardium origin of the S3 is already refuted in previous study (Van de Werf et al., 1984a). Secondly, we ignored S3 mixture with other sounds like noise and thoracic damping effect, because they do not change the general generation mechanism of S3 and its relations with hemodynamics which are the main goals of this study. The full parametrization of CircAdapt has been done before (Walmsley et al., 2015; Shahmohammadi et al., 2021) and we do believe that it is sufficient for the patient-generic model used in this study. When aiming for patient-specific simulations both sensitivity analysis and parameter identification are of key importance, needing a more complete parameter identification from experimental data for expressing the state of the model as a function of independent parameters.

## Conclusion

To the best of our knowledge, this is the first simulation study that uses an S3 generator module embedded in a complete real-time model of the cardiovascular system capable of simulations of various cardiac conditions. While the resulting model is simple, it realistically simulates S3 in both accurate and robust real-time simulations of conditions with different types and degrees of severity including HFpEF, AVR, AVS and SS under both rest and exercise.

## Data Availability

The original contributions presented in the study are included in the article; further inquiries can be directed to the corresponding author. The software used can be found here: https://github.com/pydart/S3.

## Appendix A:

### S1

#### Integrated Loudness

Integrated loudness in the present paper is calculated using a function with the same name in MATLAB software (The Math Works, Inc. MATLAB. Version 2020a). The input channels, *x*, pass through a K-weighted weighting Filter. The K-weighted filter shapes the frequency spectrum to reflect perceived loudness.

#### Relevant Steps to Estimate Integrated Loudness

1. The K-weighted channels, *y*, are divided into 0.4-s segments with 0.3-s overlap. The power (mean square) of each segment of the K-weighted channels is calculated:

$$mP_{i}=\frac{1}{w}\;\sum_{k=1}^{w}y_{i}^{2}\left[k\right]$$

• $mP_{i}$ is the momentary power of the i th segment of a channel.
• w is the segment length in samples.
2. The momentary loudness, *mL*, is computed for each segment:

$$mL_{i}=-0.691+10\;\log_{10}\left(\sum_{c=1}^{n}G_{c}\times mP_{\left(i,c\right)}\right)\;\mathrm{LUFS}$$

• $G_{c}$ is the weighting for channel c .
3. The momentary power is gated using the momentary loudness calculation:

$$\begin{array}{l} mP_{i}\to mP_{j}\\ j=\left\{i|mL_{i}\mathrm{\geq -}70\right\} \end{array}$$

4. The relative threshold, *Γ*, is computed:

$$\mathrm{\Gamma =-}0.691+10\;\log_{10}\left(\sum_{c=1}^{n}G_{c}\times l_{c}\right)-10$$

• $l_{c}$ is the mean momentary power of channel c :

$$l_{c}=\frac{1}{\left|j\right|}\sum_{j}mP_{\left(j,c\right)}$$

5. The momentary power subset, *mP*_*j*_, is gated using the relative threshold:

$$\begin{array}{l} mP_{j}\to mP_{k}\\ k=\left\{j|mP_{j}\geq \text{Γ}\right\} \end{array}$$

6. The momentary power segments are averaged:

$$P=\frac{1}{\left|k\right|}\sum_{k}mP_{k}$$

7. The integrated loudness is computed by passing the mean momentary power subset, *P*, through the Compute Loudness system:

$$\mathrm{Integrated}\;\mathrm{Loudness}\;=-0.691+10\;\log_{10}\left(\sum_{c=1}^{n}G_{c}\times P_{c}\right)\;\;\mathrm{LUFS}$$

## References

[B1] Arevalo F. Meyer E. C. MacCanon D. M. Luisada A. A. (1964). Hemodynamic correlates of the third heart sound. Am. J. Physiol. 207 (2), 319–324. 10.1152/ajplegacy.1964.207.2.319 14205341

[B4] Cowie M. R. Wood D. A. Coats A. J. S. Thompson S. G. Suresh V. Poole-Wilson P. A. (2000). Survival of patients with a new diagnosis of heart failure: A population based study. Heart 83 (5), 505–510. 10.1136/heart.83.5.505 10768897 PMC1760808

[B26] Dock W. Grandell F. Taubman F. (1955). The physiologic third heart sound: its mechanism and relation to protodiastolic gallop. Am. Heart J. 50 (3), 449–464. 10.1016/0002-8703(55)90166-6 14398637

[B5] Drzewiecki G. M. Wasicko M. J. Li J. (1991). Diastolic mechanics and the origin of the third heart sound. Ann. Biomed. Eng. 19 (6), 651–667. 10.1007/BF02368074 1781567

[B6] Folland E D. Bruce J. Kriegel W. G. H. Karl E. Hammermeister G. K. (1992). Implications of third heart sounds in patients with valvular heart disease. The veterans affairs cooperative study on valvular heart disease. N. Engl. J. Med. 327 (7), 458–462. 10.1056/NEJM199208133270703 1625735

[B7] Gazewood J D. Turner P. L. (2017). Heart failure with preserved ejection fraction: Diagnosis and management. Am. Fam. Physician 96 (9), 582–588. 29094875

[B8] Gheorghe A. G. Fuchs A. Jacobsen C. Kofoed K. F. Møgelvang R. Lynnerup N. (2019). Cardiac left ventricular myocardial tissue density, evaluated by computed tomography and autopsy. BMC Med. imaging 19 (1), 1–9. 10.1186/s12880-019-0326-4 30979363 PMC6461811

[B9] Held P. Lindberg B. Karl S. (1984). Audibility of an artificial third heart sound in relation to its frequency, amplitude, delay from the second heart sound and the experience of the observer. Am. J. Cardiol. 53 (8), 1169–1172. 10.1016/0002-9149(84)90656-8 6702698

[B2] Liu C. Springer D. Li Q. Moody B. Juan R. A. Chorro F. J. (2016). An open access database for the evaluation of heart sound algorithms Physiol. Meas. 37 (12), 2181–2213. 10.1088/0967-3334/37/12/2181 27869105 PMC7199391

[B11] Longhini C. Scorzoni D. Baracca E. Brunazzi M. C. Chirillo F. Fratti D. (1996). The mechanism of the physiologic disappearance of the third heart sound with aging. Jpn. Heart J. 37 (2), 215–226. 10.1536/ihj.37.215 8676548

[B12] Lumens J. Fan C.‐P. S. Walmsley J. Yim D. Manlhiot C. Dragulescu A. (2019). Relative impact of right ventricular electromechanical dyssynchrony versus pulmonary regurgitation on right ventricular dysfunction and exercise intolerance in patients after repair of tetralogy of Fallot. J. Am. Heart Assoc. 8, e010903–2. 10.1161/JAHA.118.010903 30651018 PMC6497336

[B13] Mann D. L. (2008). Braunwald’s heart disease: A textbook of cardiovascular medicine. 8th ed. Philadelphia: WB Saunders, 611–640.Management of heart failure patients with reduced ejection fraction

[B14] Mehta N. J. Khan I. A. (2004). Third heart sound: Genesis and clinical importance. Int. J. Cardiol. 97 (2), 183–186. 10.1016/j.ijcard.2003.05.031 15458681

[B15] Miranda W. R. Newman D. B. Geske J. B. Nishimura R. A. (2015). An auscultatory conundrum: Severe mitral stenosis with a third heart sound? Eur. Heart J. Cardiovasc. Imaging 16 (8), 918. 10.1093/ehjci/jev122 25925214

[B16] Patel R. Bushnell D. L. Sobotka P. A. (1993). Implications of an audible third heart sound in evaluating cardiac function. West. J. Med. 158 (6), 606–609. 8337855 PMC1311785

[B25] Reddy P. S. Meno F. Curtis E. I. O’Toole T. (1981). The genesis of gallop sounds: investigation by quantitative phono- and apexcardiography. Circulation 63 (4), 922–933. 10.1161/01.cir.63.4.922 7471348

[B17] Shahmohammadi M. Luo H. Westphal P. Cornelussen R. N. Prinzen F. W. Delhaas T. (2021). Hemodynamics-driven mathematical model of first and second heart sound generation. PLoS Comput. Biol. 17 (9), e1009361. 10.1371/journal.pcbi.1009361 34550969 PMC8489711

[B18] Van de Werf F. Boel A. Geboers J. Minten J. Willems J. De Geest H. (1984b). Diastolic properties of the left ventricle in normal adults and in patients with third heart sounds. Circulation 69 (6), 1070–1078. 10.1161/01.cir.69.6.1070 6713611

[B19] Van de Werf F. Minten J. Carmeliet Peter De Geest H. Kesteloot H. (1984a). The Genesis of the third and fourth heart sounds. A pressure-flow study in dogs. J. Clin. Invest. 73 (5), 1400–1407. 10.1172/JCI111344 6715543 PMC425163

[B20] van Loon T. Christian K. Cornelussen R. Reesink K. D. La Rocca H.-P. B. Delhaas T. (2020). Increased myocardial stiffness more than impaired relaxation function limits cardiac performance during exercise in heart failure with preserved ejection fraction: A virtual patient study. Eur. Heart J. - Digital Health 1 (1), 40–50. 10.1093/ehjdh/ztaa009 PMC9707905 36713963

[B21] Vancheri F. Gibson D. (1989). Relation of third and fourth heart sounds to blood velocity during left ventricular filling. Br. Heart J. 61 (2), 144–148. 10.1136/hrt.61.2.144 2923750 PMC1216631

[B22] Walmsley J. Arts T. Derval N. Bordachar P. Hubert C. Ploux S. (2015). Fast simulation of mechanical heterogeneity in the electrically asynchronous heart using the MultiPatch module. PLoS Comput. Biol. 11 (7), e1004284. 10.1371/journal.pcbi.1004284 26204520 PMC4512705

[B23] Wilken M. K. Meyers D. G. Lakshi P. A. Frank P. Y. Starke H. Starke H. (1989). Mechanism of disappearance of S3 with maturation. Am. J. Cardiol. 64 (19), 1394–1396. 10.1016/0002-9149(89)90593-6 2589213

[B24] Zhong L.-S. Guo X. -M. Xiao S.-Z. Wang D. Wu W. -Z. (2011). The third heart sound after exercise in athletes: An exploratory study. Chin. J. Physiol. 54, 219–224. 10.4077/CJP.2011.AMM049 22129819

